# Right-wing authoritarianism and stereotype-driven expectations interact in shaping intergroup trust in one-shot vs multiple-round social interactions

**DOI:** 10.1371/journal.pone.0190142

**Published:** 2017-12-28

**Authors:** Giorgia Ponsi, Maria Serena Panasiti, Salvatore Maria Aglioti, Marco Tullio Liuzza

**Affiliations:** 1 Department of Psychology, University of Rome “Sapienza”, Rome, Italy; 2 Social and Cognitive Neuroscience Laboratory, IRCCS Fondazione Santa Lucia, Rome, Italy; 3 Department of Medical and Surgical Sciences, “Magna Graecia” University of Catanzaro, Loc. Germaneto, Catanzaro; Tilburg University, NETHERLANDS

## Abstract

Trust towards unrelated individuals is often conditioned by information about previous social interactions that can be derived from either personal or vicarious experience (e.g., reputation). Intergroup stereotypes can be operationalized as expectations about other groups’ traits/attitudes/behaviors that heavily influence our behavioral predictions when interacting with them. In this study we investigated the role of perceived social dimensions of the Stereotype Content Model (SCM)–Warmth (W) and Competence (C)—in affecting trusting behavior towards different European national group members during the Trust Game. Given the well-known role of ideological attitudes in regulating stereotypes, we also measured individual differences in right-wing authoritarianism (RWA). In Experiment 1, we designed an online survey to study one-shot intergroup trust decisions by employing putative members of the European Union states which were also rated along SCM dimensions. We found that low-RWA participants’ trusting behavior was driven by perceived warmth (i.e., the dimension signaling the benevolence of social intentions) when interacting with low-C groups. In Experiment 2, we investigated the dynamics of trust in a multiple-round version of the European Trust Game. We found that in low-RWA participants trusting behavior decreased over time when interacting with high-W groups (i.e., expected to reciprocate trust), but did not change when interacting with low-W groups (i.e., expected not to reciprocate trust). Moreover, we found that high-RWA participants’ trusting behavior decreased when facing low-W groups but not high-W ones. This suggests that low-RWA individuals employ reputational priors but are also permeable to external evidence when learning about others’ trustworthiness. In contrast, high-RWA individuals kept relying on stereotypes despite contextual information. These results confirm the pivotal role played by reputational priors triggered by perceived warmth in shaping social interactions.

## Introduction

Social decisions are described as choices that influence both ourselves and other individuals, and are consequently affected by both self- and other-regarding preferences [1]. Importantly, social decisions are usually taken in interpersonal contexts that include other social agents whose mental states, intentions and future behaviors are characterized by uncertainty [2].

In humans, widespread altruism toward genetically unrelated individuals probably required the evolution of specific psychological tools [3]. One of these tools is represented by trust, that is the willingness to take the risk of helping another individual despite the possibility of not being helped back [4]. Paradigms from experimental economics (Game Theory[5]) provide an ecologically valid way to determine social preferences in the laboratory setting, and how social decisions can be influenced by dispositional and situational factors [6–8]. In particular, decisions to trust and to reciprocate trust are usually investigated by employing the Trust Game (TG[9]). The TG can be played both as single-round game in which participants take decisions only once (i.e., one-shot decisions) and as multiple-round game in which decisions are re-iterated several times. Interestingly, these variants strongly affect both optimal and actual game strategies because of the effect of potential consequences on current choices [10].

On the one hand, behavioral Game Theory [11] predicts that—when playing only once—a rational and selfish trustee will never pay back the investor. Therefore a rational investor should invest nothing in the first place [4]. Despite these assumptions, a majority of investors are prone to send some money to the trustee (approximately half of their endowment) and this trust is usually reciprocated [11]. On the other hand, during repeated social interactions, choices change continuously as knowledge about others’ trustworthiness are incrementally updated via reinforcement learning mechanisms [12]. In fact, trust towards other individuals is often conditioned by information about previous interactions; this information can be inferred from either personal experience (direct reciprocity) or vicarious experience (i.e., reputation; indirect reciprocity, for reviews see [13,14]).

The role of vicarious experience in shaping trusting behaviors has been highlighted by studies that showed how trustees’ actual behavior is not the only factor that predicts our decisions on whether to trust or not them during a TG [15,16]. For instance, trustees’ race and face emotional expressions can strongly modulate social decisions [17].

Crucially, stereotypes can be operationalized as expectations about other people’s behavior that—in turn—can heavily influence our predictions [18]. Previous findings highlighted that behavioral predictions strongly rely on inferences about traits, social categories, reputation and physical features, that guide decision-making processes when we face a person or a group [2]. Stereotype Content Model (SCM[19]) is a psychological theory that proposes the mechanisms trough which societal stereotypes are built and their relation to the actual social structure. According to the SCM, two basic dimensions of social perception—Warmth (W) and Competence (C)—are employed in order to form impressions or interpret behavior of other individuals/groups. More specifically, W is used as a social cue to predict conspecifics’ perceived intent (e.g., to help or to harm) and is related to perceived interdependence (cooperative-competitive), while C is used as an index of the ability to enact perceived intentions and is related to perceived socio-economic status [20–22]. SCM also describes how stereotype’s content generates distinct emotions felt toward different social groups (BIAS map [20]): high W-high C (admired groups, like the in-group and the aspirational groups); low W-high C (envied groups, like Asians, Jews and rich people); high W-low C (paternalized groups, like elderly and disabled); low W-low C (derogated social groups, like homeless people, poor people and immigrants).

Evidence for group-based trust emerges from data suggesting that in-group trustees are preferred over out-group ones because of the *group heuristic-based trust*, i.e. the expectation that a fellow in-group member will act in a fair and reciprocal manner [23,24]. Moreover, the degree of racial bias seems to be a modulator of economic behavior in intergroup contexts, since participants with a greater implicit bias (as measured by IAT [25]) decide to behave less fairly in the Ultimatum game (an economic game generally employed to study resources allocation and altruistic punishment behavior [26,27]) and to invest less on out-groups in the TG [28].

The present study aims to assess the role of perceived SCM social dimensions (Warmth and Competence) in making economic decisions with putative players belonging to different social groups when playing the Trust Game [9]. In fact, few studies investigated the role of SCM dimensions in economic decision-making. Competence-based trust seems to represent a well-preserved mechanism to favor high-competence/status members. Consistently, a previous study found that low-C group members (Southern Italians) invested more money on high-C out-group members (Northern Italians) than on low-C in-group ones [29]. This is not surprising, considering that the reliance on status when deciding to trust is already present in early childhood [30] and affects the way resources are allocated in real-world financial interactions [31]. It is worth noting, however, that warmth-related cues are often prioritized respect to competence-related cues when taking trust-related investment decisions, supporting the primacy of warmth hypothesis [32,33].

SCM social dimensions do not seem to be universally employed in the same way, since their use is heavily affected by individual differences. Recently, we reported a differential employment of these two social dimensions during a categorization task in participants who tend to include (by using a warmth-centered decision criterion) or exclude (by using a competence-centered decision criterion) new members from their in-group [34,35]. Moreover, using the Temptation to Lie Card Game [36–38] we showed that perceived warmth and competence differentially influenced the tendency to deceive others and the impact of reputation on deception [39]. In particular, we found that perceived warmth decreased the amount of self-gain lies while perceived competence increased it and that the impact of reputation on deception was higher when participants were playing with participants perceived low in both dimensions.

A recent study showed that the association between status and competence—but not status and warmth—is moderated by ideological beliefs and attitudes toward inequality [40], suggesting that political ideology might affect not only the way we use stereotypes, but the content of the stereotypes that we attach on different groups. This suggests that when investigating the relationship between stereotypes and perceived SCM social dimensions, it is crucial to take into consideration also individual differences in political ideology. Oldmeadow and Fiske [40] investigated the interaction between SCM and Belief in a Just World [41] and Social Dominance Orientation (SDO[42]), but not Right-Wing Authoritarianism (RWA[43]), which, together with SDO trait, represents a core aspect of political conservatism and a predictor of intergroup bias [44–46]. The relationship among individual differences in RWA, SCM dimensions and stereotyping has not been extensively investigated in the previous studies.

Crucially, RWA is especially interesting since it is an ideological attitude strongly related to prejudice directed towards other groups [47]. Right-wing authoritarians are in fact characterized by (i) higher level of prejudice and conservative political attitudes [48], (ii) willingness to reward group-consistent behaviors and punish inconsistent ones [49], (iii) readiness to be hostile and punitive towards threatening or unconventional others [43], (iv) greater reliance on heuristic processing and rigidity in categorization [49,50], (v) intolerance to ambiguity [51], (vi) low levels of openness to experience, high levels of conscientiousness and neuroticism [52], and (vii) proneness to established authorities and a strong adherence to societal conventions [43].

Intergroup responses often require self-regulation mechanisms in order to inhibit biases; in particular, *prejudice control* refers to responding with egalitarian intentions despite the activation of automatic stereotypes. Social psychology research highlighted that low-prejudiced people are motivated to exert control on their intergroup responses, whereas highly-prejudiced people are not [53]. On the political spectrum, liberals are usually better than conservatives at responding without prejudice [54,55], probably because egalitarian ideology motivates them to downplay their bias, especially towards low status/low competence groups.

The present study aims to assess the role of perceived SCM social dimensions (Warmth and Competence) and of ideological attitudes (Right-Wing Authoritarianism) in making economic decisions with putative players belonging to different European national groups when playing the Trust Game [9]. Since the variant (i.e., one-shot *vs* multiple-rounds) of the game profoundly affects the strategies employed by participants, we employed both in two different experiments. In Experiment 1 we employed a one-shot version of the Trust Game played with trustees from other European Union countries, in order to test how trust is generally affected by intergroup stereotypes about warmth and competence without any behavioral feedback from the trustees. We hypothesized that subjective perception of SCM social dimensions (W and C) would be able to explain differences in participants’ trusting behavior. Since both warmth and competence represent pivotal dimensions of social cognition [56], we expected that groups perceived high in both dimensions would be the more trusted. Concerning individual differences in RWA, we expected that low-RWA participants—considering their higher egalitarian motivation—would tend to regulate their stereotypes more than high-RWA ones, in agreement with the role of ideological attitudes in the stereotype inhibition process [55,57]. Since during prolonged social interactions decision strategies are updated over time depending on information about others’ trustworthiness [12], in Experiment 2, we employed a multiple-round version of the Trust Game, in order to test whether stereotypes about warmth and competence would affect intergroup trust also during interactions characterized by neutral behavioral feedbacks from putative players from Italy (ingroup), Germany (low-W, high-C outgroup) and Greece (high-W, low-C outgroup). In keeping with studies showing that both authoritarianism and conservatism involve resistance to change [46], we expected that low-RWA players would adjust their behavioral strategy according to others’ feedback, while high-RWA participants would tend not to learn from the neutral reciprocation feedback and would rely on their prejudices more than low-RWA participants. Materials, data and analysis scripts of both experiments are available on Open Science Framework: https://osf.io/d8uen/?view_only=d896c19f753c4978ba8ffbb9b22eb0bb.

## Material and methods

### Experiment 1

#### Participants

64 Italian participants (37 male, age range 18–52 years, M = 31.84, SD = 7.45) were recruited by posting an invitation to complete an online experiment pictured on CrowdFlower website. Crowdsourcing platforms like CrowdFlower and Amazon Mechanical Turk represent viable alternatives for high quality data collection [58–62]. A control question (*If you are correctly reading this question*, *instead of using the rating scale*, *please write down in the text box “Other”*:*“I have carefully read*”) was included in order to exclude participants who were not sufficiently focused on the experiment; we excluded 14 participants for this reason. The data of 50 participants (27 male, age range 18–52 years, M = 31.86, SD = 8.05) were used for the analyses. Sample size was not determined by power analysis but it was similar to that of other studies investigating SCM dimensions and decision-making [32,33]. Participants were informed that they would receive a basic compensation of €1 for their time plus a variable compensation ranging from €2 to €3 depending on their decisions; they were reimbursed accordingly (M = 2.81€, S.D. = 0.39). The study was approved by the local ethics committee of Istituto di Ricovero e Cura a Carattere Scientifico Fondazione Santa Lucia, and was performed in accordance with ethical standards of the 1964 Declaration of Helsinki after obtaining written informed consent from each participant.

#### Task and design

**Fig 1:**
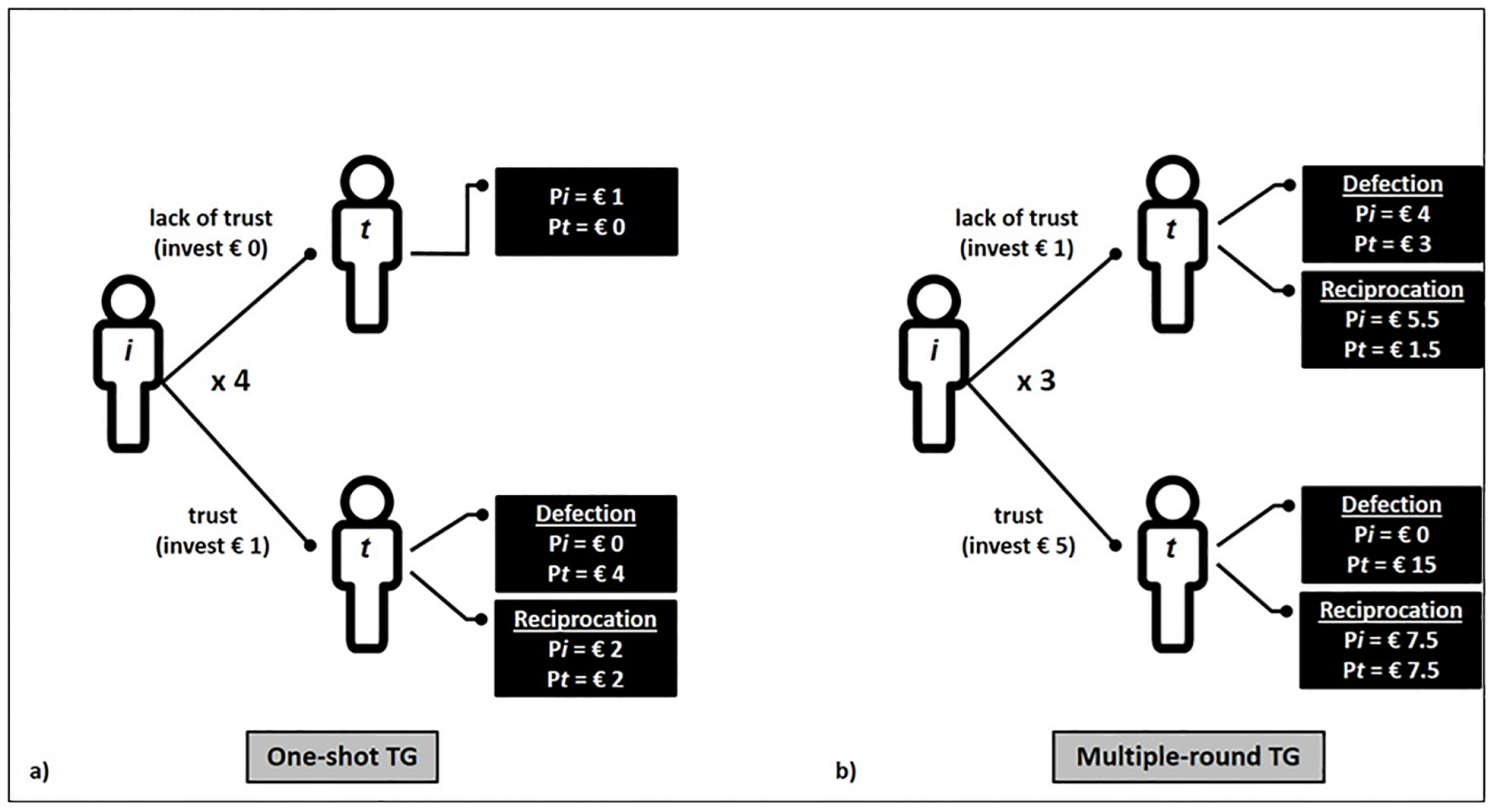
(A) Trust Game decision tree representing all the possible outcomes of the one-shot version of the game (Experiment 1). (B) Trust Game decision tree representing all the possible outcomes of the multiple-round version of the game (Experiment 2). P*i* is the payoff of the participants who always played the role of investor, P*t* is the payoff of the other player who always plays the role of trustee.

The survey was composed of 28 consecutive rounds of the Trust Game (TG[9]) with different counterparts belonging to the 28 European Union Member States. In our adapted version (see Fig 1A), during each trial the participant (i.e., the investor) was endowed with €1 and could decide whether to invest €0 or €1 in the trustee. Once invested, money was multiplied by four, becoming an amount of €0 or €4, depending on the investor’s initial choice. Participants were informed that the trustees previously made a series of hypothetical choices recorded in our database; in particular, they recorded what they would have done if they had received an investment of €1. The trustee had to decide whether to keep for him/herself the entire amount of money or to reciprocate trust by returning half of the sum (e.g., €2 in the case of an original investment of €1). The decision to invest €1 represents a risky choice: if the investor made an investment of €1 and the trustee returns half of the sum, both players end up with a payoff higher than the original endowment; on the other hand, if the trustee abuses the trust, the investor gains nothing. At the beginning, participants were presented with detailed instructions (see Supporting Information, S1 Text, Experiment 1 Instructions). During each trial of the economic game, participants were presented with the flag image (size: 150 X 101 pixel) of the specific country (e.g., France) together with the following reminder: “*In the case that this trial will be extracted for determining your additional compensation*, *remember that if you will decide to invest €0*, *you will gain €1*, *while if you will decide to invest €1*, *the gain will depend on the trustee*: *if he/she will reciprocate your trust*, *you will gain €2*, *while if he/she will decide to not reciprocate your trust*, *you will gain €0*. *How much do you want to invest in a person coming from FRANCE*?”. At this point, participants had to choose between the two forced alternatives of €0 and €1. Importantly, they were informed that the extracted trial would be employed to determine the final compensation for them and the player they were interacting with (implying real monetary consequences for both of them). Unbeknown to the participants, the game took place against a PC device. For a detailed view of the TG decision tree, please see Fig 1A. After the Trust Game, participants rated people coming from the 28 European Union Member States along the warmth (“*How warm* (*i*.*e*., *friendly*, *likeable*) *do you think a person coming from ___ is*?”) and competence (“*How competent* (*i*.*e*., *able*, *rational and dominant) do you think a person coming from ___ is*?”) dimensions on 16-points Likert-type scales. Finally, they completed the RWA Scale (Right-Wing Authoritarianism Scale[43,63,64]).

In order to determine the final compensation, we did not extract a random trial but we decided to insert an additional 29^th^ trial in which participants played against a hypothetical participant coming from Switzerland (a European Country that does not belong to the European Union). 52 of the 64 participants of the entire sample (and 42 of the 50 participants of the analyzed sample) decided to invest in the Swiss person, so they gained €3, while the participants who decided to not invest in the Swiss person gained €2.

#### Data analysis

Since the use of ANOVA on frequencies is inappropriate [65], analyses on investments were performed using logit mixed models. Ratings on liking and friendliness were averaged into a unique Warmth measure. Ratings on Competence were obtained by averaging competence, rationality and dominance (since the latter has been found constantly correlated to competence [19,22,66]) (see Supporting Information, S1 Table, for descriptive information about warmth and competence ratings). The mean score of the 10 items RWA Scale ([63,64]; Cronbach’s α = .77) was of 3.85 (SD = .84), on a 7-points Likert-type scale.

### Results

#### One-shot TG

To estimate the intercept-only model, we fitted our data in a generalized linear model by the *glm* function in R [67]. Then, we ran Generalized Mixed Linear-Effects (MLE, binomial family) by using *glmer* function from the *lme4* R package [67,68], with one dependent variable (Invest/Not-Invest decision) and three predictor-independent covariates (Warmth, Competence, RWA) with all the reciprocal interactions. Participants were entered as random factors and fixed effects were also modeled as random slopes over participants [69]. We found that both Warmth and Competence represent significant predictors of trusting behavior (Warmth: χ^2^(1) = 21.37, *p* < .001; Competence: χ^2^(1) = 28.62, *p* < .001). Also the two-way interaction between warmth and competence is significant (χ^2^(1) = 10.66, *p* = .001). Despite RWA predictor itself does not reach significance (RWA: χ^2^(1) = 0.40, *p* = .525), its interaction with warmth (χ^2^(1) = 4.94, *p* = .026) and competence (χ^2^(1) = 5.38, *p* = .020) seems to significantly predict participants’ investment behavior in the Trust Game. All these main effects and two-way interactions are qualified by a significant three-way interaction among warmth, competence and RWA (χ^2^(1) = 8.25,*p* = .004).

We also tested the simple effects of the triple interaction in a regression model (including also the random effects) by means of the function simple slopes included in the R package *reghelper*. The simple slopes for the association between warmth and trusting behavior were tested for low (-1 SD below the mean) and high (+1 SD above the mean) levels of the two other continuous variables (competence and RWA).

**Fig 2:**
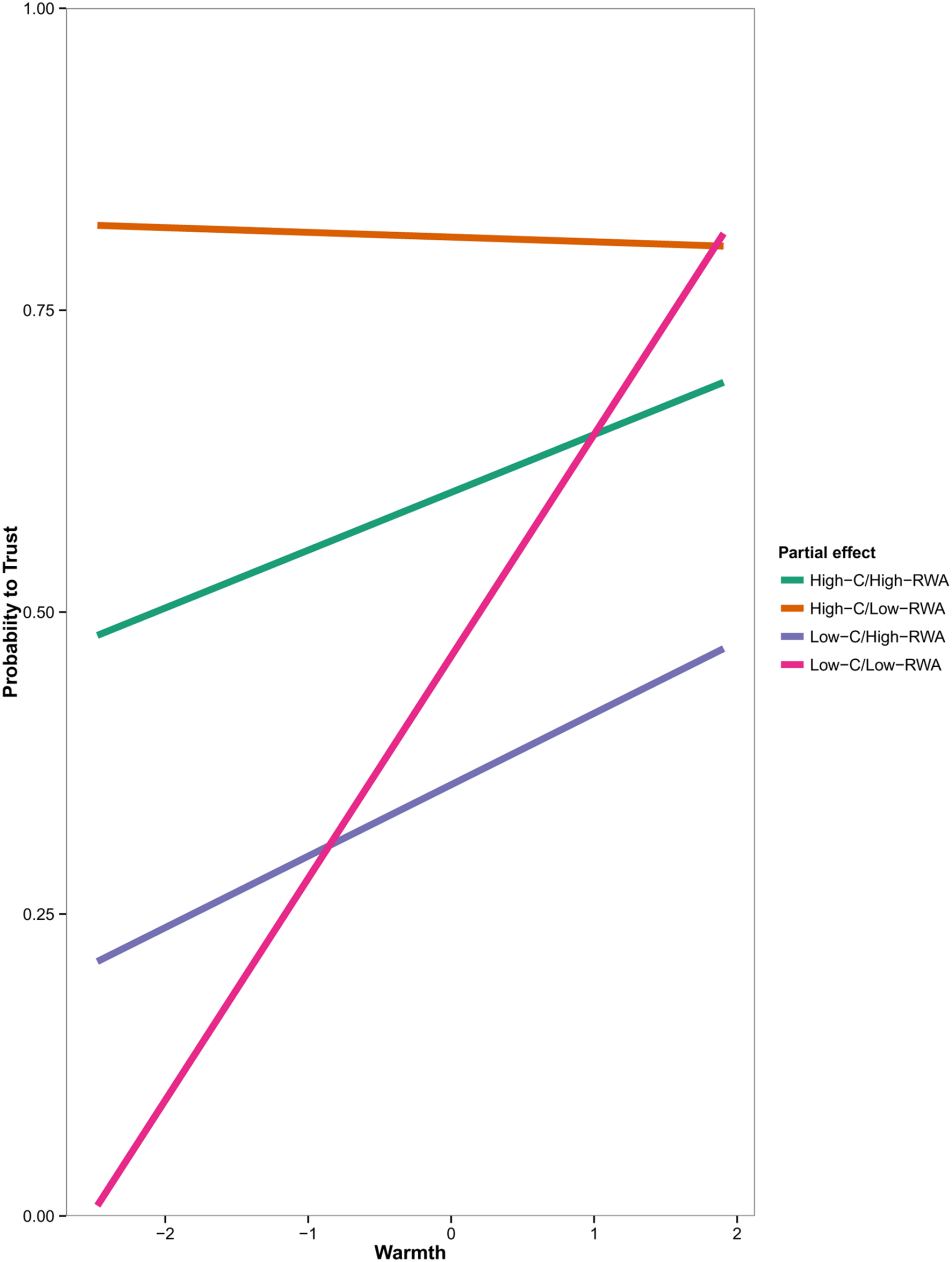
Predicted probability to invest in the Trust Game when considering the three-way interaction among Warmth, Competence and RWA. Predictions are based on estimates derived from the simple slopes analysis.

The analysis showed that when both groups’ perceived competence and investor’s RWA score are low, there is a significant relationship between warmth and trusting behavior (b = 1.31, SE = .27, t = 4.83, upper CI = 1.84, lower CI = .78, see Fig 2, low-C/low-RWA). The combinations high-C/high-RWA (b = .205, SE = .16, t = 1.31, upper CI = .51, lower CI = -.10), high-C/low-RWA (b = -.03, SE = .21, t = -.16, upper CI = .37, lower CI = -.44) and low-C/high-RWA (b = .27, SE = .23, t = 1.17, upper CI = .72, lower CI = -.18) did not entail significant effects.

In particular, the three-way interaction shows that, when playing against low-C groups, low-RWA participants were driven by perceived warmth: they tended to trust high-W/low-C members and to distrust low-W/low-C ones (see Fig 2, Low-C/Low-RWA). This signals that low-RWA participants are particularly sensitive to warmth dimension (at least when interacting with low-C social groups), in agreement with its role in predicting the goodness of others’ intentions. No behavioral pattern was significant in participants who scored higher in RWA (see Fig 2).

### Discussion

In Experiment 1 we investigated the role of perceived SCM social dimensions (Warmth and Competence) and of ideological attitudes (Right-Wing Authoritarianism) in making economic decisions with putative players belonging to European Union national countries when playing a single-round version of the Trust Game [9]. Our findings suggest that the perceived social dimensions described by SCM [19] are able to impact participants’ investment behavior when taking one-shot trust decisions.

We found that participants were more likely to invest in trustees from national groups perceived high in warmth and competence (in agreement with SCM predictions about the role of warmth and competence in inter-group social cognition; [19,56]). This finding is also in line with a recent work reporting that individuals have shared stereotypes in terms of expected cooperation for interaction partners from different countries and that these stereotype-driven expectations represent the strongest predictor of participants’ actual cooperation [70]. In addition, we showed that individual differences in Right-Wing Authoritarianism interacted with SCM social dimensions in shaping intergroup decision-making. On the one hand, we found that low-RWA participants’ investment behavior was guided by perceived warmth dimension, but only when facing low-C groups (i.e., high-W/low-C social groups). On the other hand, low-RWA participants seem to adopt stereotypical behavior when facing members of low-W/low-C social groups, by investing less money on them. This finding shows that when low-RWA participants face low-C groups’ members, trust is enhanced only when the trustees are characterized by a positive reputational prior (i.e., high ratings of W that signal good social intentions).

Contrary to our expectations, we did not find evidence of stereotypical behavior in high-RWA participants. This is in line with recent findings suggesting that conservatives do not differ from liberals when considering the amount of prejudice toward dissimilar social targets [71]. Alternatively, it has been shown that the relationship between authoritarianism and the expression of prejudice might be determined by the presence of an authority’s explicit instruction [72], which in our case was not present. Thus, it is entirely possible that stereotypes regarding the other player were not sufficient to trigger any discriminatory behavior in high-RWA participants.

In this first experiment, we employed an online survey because we aimed at testing whether the pattern of immediate one-shot trust decisions toward players of different European Union national countries would follow the intergroup stereotypes defined by the SCM [19]. In the second study, we aimed to test how intergroup stereotypes could drive participants’ investment behavior over time when provided with information about the outcome of other players’ decisions (i.e., reciprocation *vs* defection). So, in order to broaden the findings of Experiment 1, we designed a multiple-round (i.e., decisions are re-iterated several times) onsite behavioral experiment to further investigate intergroup trust behavior and the role of authoritarianism by employing a representative sub-sample of putative players of the European Union member states. In particular, we implemented in the Trust Game a neutral 50% reciprocation rate (independent from group membership), in order to test whether trust decisions are driven by investors’ biased interpretation of group members’ reciprocation behavior.

Our predictions were that high-RWA participants would tend not to adjust their behavioral strategy according to other players’ actual behavior and would hold to their stereotypes more than low-RWA participants. In addition, since Experiment 1 showed that in low-RWA participants one-shot trust decisions were driven by perceived warmth (especially when interacting with low-C group members), we expected that stereotypes about warmth would affect intergroup trust also during prolonged social interactions characterized by neutral behavioral feedbacks from the other players. In particular, we hypothesized that expectations about reciprocation would be influenced by perceptions of warmth: in fact, according to SCM [19,56], low-W group members are more likely to be expected to defect (negative prior) and high-W members are more likely to be expected to reciprocate (positive prior).

### Experiment 2

The present experiment aims to assess the role of perceived SCM social dimensions (Warmth and Competence) and of ideological attitudes (Right-Wing Authoritarianism) in making economic decisions with putative players belonging to different European national groups when playing a multiple-round version of the Trust Game [9]. Since this was an onsite behavioral experiment we reduced the number of European countries involved in the experiment in order to make the cover story believable. In more detail, we selected Italians and two groups that were among the key characters of the recent European crisis: the “cicadas” Greeks and the “ants” Germans [73], that were supposed to be perceived either high in warmth (the Greeks) or in competence (the Germans), respectively.

#### Participants

44 students (14 male; age range 19–39 years, M = 24.66, SD = 3.31) of the University of Rome “Sapienza” voluntarily took part in the experiment. All of them were Italian, healthy, naïve to the purposes of the study, and had normal or corrected-to-normal vision. Sample size was not determined by power analysis but it was similar to that of other studies investigating SCM dimensions and decision-making [32,33]. Participants were informed that they would receive a compensation of €5 for their participation, plus the money gained in one randomly extracted round of the game; they were reimbursed with an amount between 5 and 12.50 euro (M = 9.81€, SD = 2.14). The study was approved by the local ethics committee of Istituto di Ricovero e Cura a Carattere Scientifico Fondazione Santa Lucia, and was performed in accordance with ethical standards of the 1964 Declaration of Helsinki after obtaining written informed consent from each participant.

#### Task and design

**Fig 3:**
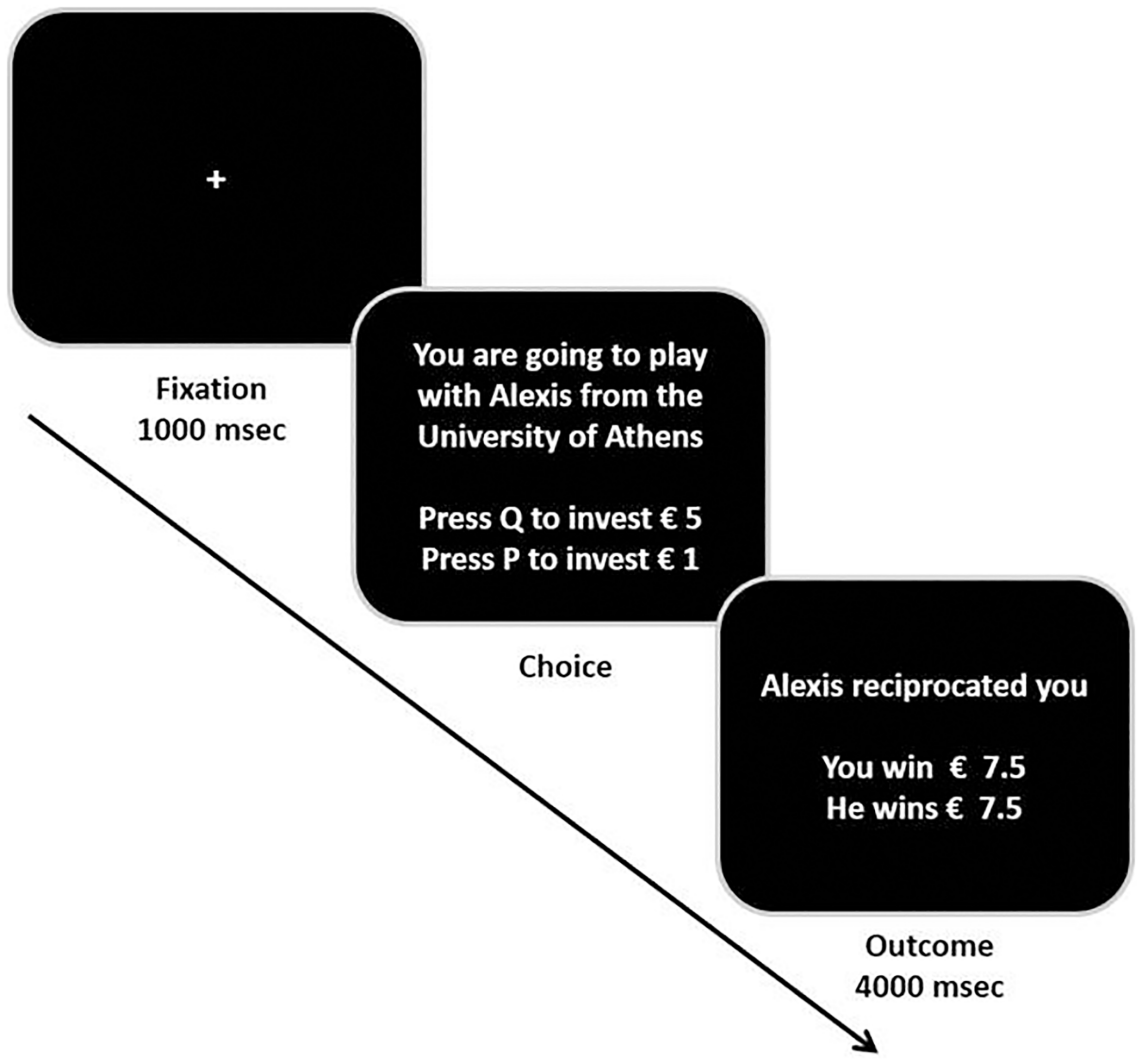
Exemplar single trial of the multiple round-version of the TG.

At the beginning of the experiment, participants were told that they were to take part in a study ran in partnership with the local Italian University, with a Greek and a German University. The task was based on the Trust Game [9]. In our adapted version (see Figs 1B and 3), the participant (i.e., the investor) was endowed with €5 before starting a round composed of two stages: in stage 1, the investor had to decide how much of the €5 to invest in a partner (i.e., the trustee), making a forced choice between €1 and €5; after the transfer, the money was multiplied by three, becoming an amount of €3 or €15, depending on the investor’s initial choice. In stage 2, the trustee had to decide whether to return half of the sum (€1.5 or € 7.5) to the investor or to keep for himself the entire amount of money (€3 or €15). Participants were informed that the trustees previously made a series of hypothetical choices recorded in our database; in particular, they recorded what they would have done if they had received an investment of €1 or €5. The decision to invest €5 represents a risky choice: if the investor made an investment of €5 and the trustee returns half of the sum, both players end up with a payoff higher than the original endowment; on the other hand, if the trustee abuses the trust, the investor gains nothing.

In order to acquaint with the task, participants were required to have a 6-trial practice session, in which they played as both investor and trustee; these trials were anonymous, in that they did not know the name nor the nationality and the gender of the player they were interacting with. At the beginning of the experimental task, participants were presented with detailed instructions (see Supporting Information, S2 Text, Experiment 2 Instructions). Afterwards, they were reminded that one round would be randomly extracted in order to determine the final compensation for them and the player they were interacting with (implying real monetary consequences for both of them).

During the experiment, they played in a multiple round version of the TG, always in the role of investor, against trustees putatively from: Italy, Greece and Germany. During each trial, they could see the other player’s name and University (e.g., Alexis—University of Athens) and they had to press the key “Q” in order to invest €5 and the key “P” to invest €1 (the keys were assigned to both decisions in a counterbalanced order). The TG was composed by a total of 180 consecutive rounds with different counterparts (i.e., 60 rounds against Italians, 60 rounds against Greeks and 60 rounds against Germans; for each nation, 30 rounds against males and 30 against females). Experimental trials were presented to participants in a fully randomized order. Unbeknown to the participants, the game took place against a PC device, which was programmed to reciprocate them 50% of time, independently of the nationality and gender of the current player. The TG was presented using E-Prime software 1.2. [74]. After a short break, participants completed the RWA Scale (Right-Wing Authoritarianism Scale; [43,63,64]. One week after the on-site experiment, participants were sent an online survey in which they had to rate their perceived stereotypes regarding different national groups along several dimensions (friendliness, liking, competence, rationality, dominance) on 16-points Likert-type scales.

#### Data analysis

We discarded the responses for which the individual RTs fell below the 5^th^ and beyond the 95^th^ percentile values (computed on the entire sample of participants) respectively, because we reasoned that during these trials participants either paid too little attention or engaged too much time in deliberating. Ratings on liking and friendliness were averaged into a unique Warmth measure, as we did for Competence by averaging competence, rationality and dominance [19,22,66] (see Supporting Information, S3 Text and S1 Fig for additional analyses on warmth and competence ratings). The mean score of the 10 items RWA Scale ([63,64]; Cronbach’s α = .81) was of 3.03 (SD = 0.95), on a 7-points Likert-type scale. Crucially, RWA score positively correlates with Political Orientation, measured through a Likert-type self-report ranging from 1 (extremely left-wing) to 7 (extremely right-wing) (r = .44, p = .0003).

### Results

#### Multiple-round TG

First, we built a null model by fitting our data in a generalized linear model by the *glm* function in R [67], having only the constant as the fixed effect. In order to account for the subjects’ intercepts as a random variable, we ran Generalized Mixed Linear-Effects (MLE, binomial family) by using *glmer* function from the lme4 R package [67,68]. We modeled one dependent binomial variable (Invest/Not-Invest decision) and four predictors (Warmth, Competence, RWA, Trial) in the model. Participants were entered as random factors and fixed effects were also modeled as random slopes over participants [69]. We also tested all the possible covariates’ reciprocal interactions.

We found a main effect of trial, showing that trust behavior tended to decrease during the social interaction (χ^2^(1) = 10.34, *p* = .001), and a main effect of RWA (χ^2^(1) = 9.29, *p* = .002), showing that the higher the scores on the RWA scale, the lower the probability to trust others during the multiple-round TG. We also found a two-way interaction between trial and competence (χ^2^(1) = 7.74, *p* = .005). Moreover, this model showed that the investment probability was entirely explained by the significant three-way interaction effect among Warmth, RWA and trial number (χ^2^(1) = 5.82, *p* = .028). We focused on the higher order triple interaction.

We also tested the simple effects of the triple interaction in a regression model (including also the random effects) by means of the function simple_slopes included in the R package reghelper. The simple slopes for the association between trial and trusting behavior were tested for low (-1 SD below the mean) and high (+1 SD above the mean) levels of the two other continuous variables (warmth and RWA).

The analysis showed that the combinations High-W/Low-RWA (b = -.02, SE = .01, t = -2.61, upper CI = -.01, lower CI = -.03) and Low-W/High-RWA (b = -.02, SE = .01, t = -2.51, upper CI = -.01, lower CI = -.03) both entailed significant results. On the contrary, High-W/High-RWA (b = -.01, SE = .01, t = -1.92, upper CI = .01, lower CI = -.02) and Low-W/Low-RWA (b = -.01, SE = .01, t = -.75, upper CI = .01, lower CI = -.02) combinations were not significant.

**Fig 4:**
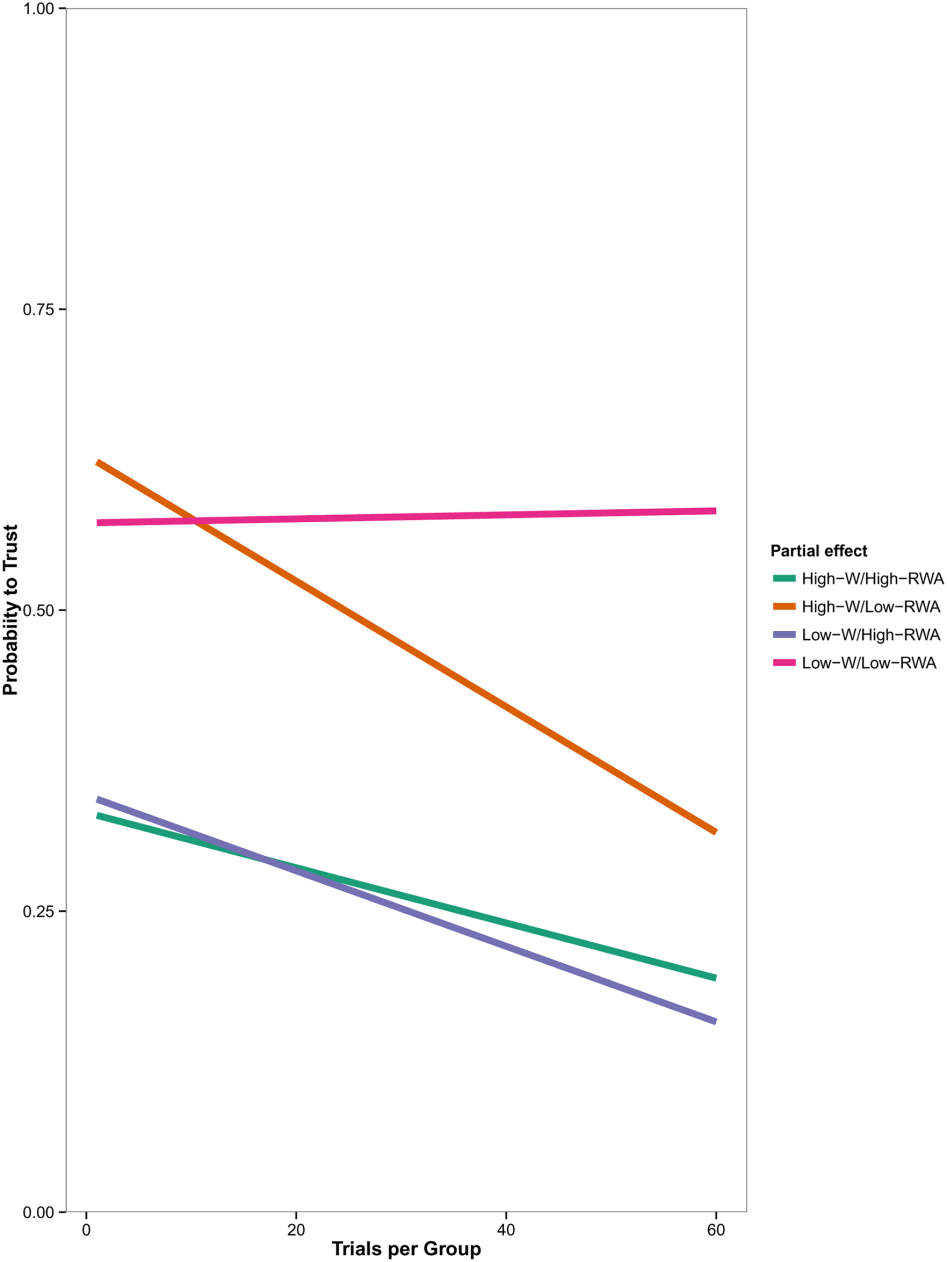
Predicted probability to invest in the Trust Game when considering the three-way interaction effect among Warmth, Trial and RWA. Predictions are based on estimates derived from simple slopes analysis.

Results show that low-RWA participants seem to initially trust more the representatives of national groups rated high in warmth (irrespectively of competence/status). This kind of positive bias triggered by warmth is in partial agreement with the findings of Experiment 1, where the investment behavior of participants toward low-C groups was driven by perceived warmth (i.e., the more the game partners were rated as warm, the more low-RWA participants invested money on them). However, as the economical interactions with the high-W partners go on—showing that they only reciprocate them 50% of time—low-RWA participants’ investment rate tends to progressively decrease over time (see Fig 4, High-W/Low-RWA). Differently, this seems not to happen for the low-W group members, since low-RWA participants trust behavior toward them seems not to vary over time (see Fig 4, Low-W/Low-RWA). Differently, as the social interactions with the low-W partners went on, high-RWA participants’ trusting behavior tended to decrease over time (see Fig 4, Low-W/High-RWA; even if also at the beginning of the Trust game, investment rate was inferior to 50% probability). Interestingly, high-RWA participants did not adjust their behavioral pattern over time when facing high-W members (Fig 4, High-W/High-RWA).

### Discussion

In Experiment 2 we tested whether the perceived social dimensions of the SCM [19] played a role in modulating intergroup bias during a multiple-round version of the Trust Game [9], in which Italian participants interacted with putative members of three different national groups (Italians, Germans and Greeks). We also explored the role of ideological attitudes (RWA) in affecting intergroup trust behavior.

Results suggest that participants tended to trust less others over time and also that the higher the scores on the RWA scale, the lower the probability to trust other players. Moreover, we found out that low- and high-RWA participants employed different behavioral strategies during the multi-round Trust Game: low-RWA participants’ investment rate toward high-W and high-RWA participants’ investment rate toward low-W partners tended to progressively decrease over time. Low-RWA participants’ behavioral pattern probably has something to do with different intergroup expectations about trustworthiness and reciprocation: low-W groups are supposedly expected to not reciprocate back the invested amount of money (since low ratings of warmth are usually associated to bad interpersonal intentions), while high-W groups are supposedly expected to reciprocate back the invested amount of money (since high ratings of warmth are usually associated to good interpersonal intentions). It seems that social expectations driven by warmth stereotype and adjustment to others’ reciprocation behavior together with the willingness to regulate (or not) pre-existing intergroup stereotypes likely interact with one another in determining the behavioral pattern of individuals. In fact, low-RWA’s egalitarian ideological attitudes seem to aid coping with the automatic reliance on group-related stereotypes by assuming exactly the opposite pattern of behavior (i.e., they do not discriminate social groups expected to not reciprocate them, as low-W ones); interestingly, the same participants tend to strongly react to defections coming from members of social groups supposed to reciprocate them, as high-W ones. The presence of differential discriminatory tendencies (present for high-W trustees but not for low-W ones) in low-RWA participants could also be due to differences in stereotype content towards the different social groups and/or to the stereotype endorsement degree towards them. Further, also high-RWA participants showed a decrease in trusting behavior over time when interacting with low-W social groups’ members, but here the behavioral adjustment seems to be less dramatic than in low-RWA participants (i.e. trusting rate was already less than 50% at the beginning of the game). High-RWA participants’ trusting behavior seemed to follow the social expectations driven by the warmth stereotype: in fact, over time they started investing less in low-W groups (i.e. who were expected not to reciprocate). Importantly, this kind of behavioral adjustment is not present when high-RWA individuals interact with high-W groups (i.e. who were expected to reciprocate). This suggests that in the presence of a positive prior, the 50% reciprocation rate is not sufficient to trigger an adjustment in their trusting behavior.

On the one hand, our findings suggest that low-RWA participants adjusted their trusting behavior to the context: despite the positive prior associated to high-W social groups, they started to invest less on them because of their actual reciprocation behavior. On the other hand, high-RWA participants adjusted their trusting behavior to the expectations triggered by the warmth stereotype: despite the same 50% reciprocation behavior, they went on investing on high-W players but they started to invest less on low-W ones. This behavioral strategy might be explained by authoritarians’ cognitive features, as a greater reliance on heuristic processing [50] and closeness to experience [52]. Consistently with these findings, Republicans have been recently found to not adjust their trust behavior to different partisan identities [75] and to show greater persistence in habitual response patterns, as indexed by neural markers of sensitivity to conflict like Error Related Negativity (ERN; [54]).

## General discussion

In this study, we employed two modified versions of the Trust Game [9] to test whether the perceived social dimensions of the SCM [19] and individual differences in ideological attitude (i.e., Right-Wing Authoritarianism; [43]) played a role in modulating intergroup trust in one-shot and multiple-round interactions. We found two interesting patterns of results: one related to general trust behavior and the other related to how trust is modulated by participants’ ideological attitude.

Focusing on SCM dimensions [19] and decision-making, we found mixed evidence in the literature. Trifiletti and Capozza (2011) pointed out that competence dimension drives the decision to invest in the Trust Game (i.e., social groups characterized by the high-C stereotype are trusted more), at least in low-C group representatives. Conversely, studies suggested that trust decisions in interpersonal contexts are mainly guided by warmth dimension [32,33]. On the one hand, in Experiment 1 we found that both perceived social dimensions (W and C) were taken into account when deciding to trust someone else during one-shot decisions. On the other hand, findings from Experiment 2 seem to suggest the existence of a relationship between warmth and trust—moderated by RWA—in prolonged social interactions characterized by neutral behavioral feedbacks.

Few studies investigated how ideology affects social decision-making. Anderson and colleagues found that—in iniquitous settings—liberals seem to trust more other players and to reciprocate them back more than conservative participants [76]. Further, a recent study shows that Republicans do not adjust their trusting behavior correspondingly to the different partisan identities, while Democrats trust more fellows of their own political party [75]. According to the authors, this behavioral pattern is driven by beliefs about partner trustworthiness—reflecting in turn stereotypes about the fellows of the other political party—that are not easily subject to change [75].

Two key results emerged from our experiments, both suggesting that warmth-related judgments heavily impact trust behavior. Firstly, in Experiment 1, low-RWA participants’ investment behavior was guided by perceived warmth, but only when facing low competence groups. In fact, participants trusted social groups rated high in warmth (i.e., high-W/low-C) but they did not invest in social groups rated low in warmth (i.e., low-W/low-C). This pattern of results suggests that trusting behavior seems to be driven by perceived (good versus bad) interpersonal intentions signaled by perceived warmth. Probably the lack of investment toward low-W/low-C social groups derives from the negative prior (i.e., lower probability to reciprocate) associated to those players. Also, fairness concerns are even more salient in the one-shot version of Trust Game (since participants had only a single interaction per group, so there is an higher risk of fraud), and this probably explains why low-RWA participants did not invest in them. In agreement, recent theories show that also liberals show similar levels of intolerance and prejudice as conservatives, especially towards dissimilar and threatening groups [77–80]. Further, this behavioral pattern might also be driven by stronger concerns about others’ fairness that characterize liberal participants [81–84] and made even more salient in the context of the one-shot version of the Trust Game.

Secondly, in Experiment 2, we found that RWA level and social groups’ perceived warmth interacted in modulating decision-making over time. In fact, we employed the multiple-round version of the Trust Game in order to test whether stereotype-driven expectations toward three different social groups (Italians, Germans and Greeks) modulated trusting behavior during repeated interactions over time. Concerning ideological attitude, we found that RWA level significantly impacts the proneness to trust other players: the higher the RWA scores, the lower the probability to trust others. We also found that low-RWA participants are initially characterized by a positive bias towards national groups’ representatives rated high in warmth (in line with liberals’ preference for warm candidates in the context of political elections [85]), but as the social interactions go on, their trusting behavior toward high-W partners tended to progressively decrease over time; interestingly the investment rate toward low-W partners (who would supposedly be perceived as less cooperative and more competitive) remain quite stable during the game. We interpreted these unexpected findings by taking into account (i) low-prejudiced individuals’ motivation to exert control on their intergroup responses [53], (ii) social expectations triggered by the warmth stereotype, (iii) the stronger permeability of low-prejudiced individuals to new evidences coming from the actual social interaction and (iv) recent theoretical developments about liberals intolerance towards dissimilar groups [77–79].

This seems to be also in line with recent neuroscientific findings showing that reputational priors (i.e., information about individualistic versus cooperative counterpart) overcome partner’s actual behavior in driving decisions to trust [15,16]. Further, reciprocity expectation even modulate brain electrical activity during the TG: promise-breaking induces larger Feedback Related Negativity (FRN, an event-related potential usually evoked by unexpected outcomes) responses to reward and non-reward discrepancies [86] and a reduced reward positivity to loss feedbacks preceded by trusting decision indexes that lack of reciprocation is unexpected by the trustor [87]. This is probably due to a sort of social expectancy violation mechanism, whose existence seems to be supported by neuroscientific evidence indicating that the brain considers social inconsistencies in a similar way to prediction errors [88–91]. In particular, the update in the behavioral strategy of low-RWA participants could be due to reward prediction error mechanisms: probably, low-RWA participants started investing more in high-W groups than low-W ones because of social expectations. Then, after they realized that reciprocation rate was around 50%, their investments dropped for high-W groups (since investment rate was higher than 50%) but not for low-W ones (since investment rate was already around 50%). In addition, low-RWA participants’ behavior might be explained as a sort of Black Sheep Effect [92] related to perceptions of warmth: in the case of deviant behavior, despite the well-known in-group favoritism phenomenon [93], in-group members are derogated more than out-group ones and are more strongly punished when violating fairness norms [94]. A similar paradoxical phenomenon emerges in moral dilemmas describing the sacrifice of White versus Black men, since White liberals decide to sacrifice more likely the White man (Studies 1a and 1b) [95]. Probably, as Uhlmann and colleagues point out, antipathy toward racial bias and the associated sensitivity to racial inequality issues both drive liberals’ decisions [95]. This is in agreement with findings showing that fairness motives are especially grave for political liberals [96] and that conservatives tend to tolerate social inequality better than liberals [46].

More importantly, low-RWA participants seem more permeable to new evidences coming from the actual social interaction respect than high-RWA ones, who showed a trusting behavior consistent with the expectation triggered by the warmth stereotype. In fact, high-RWA participants showed a decrease in trusting behavior toward low-W groups’ representatives (i.e. players stereotyped as having bad interpersonal intentions) but not toward high-W ones (i.e. players stereotyped as having good interpersonal intentions). Interestingly, the neutral 50% reciprocation rate is not sufficient to trigger an adjustment in high-RWA behavior toward the players they expect to behave fairly. Consequently, high-RWA players seem more resistant to external evidences when playing the Trust Game; this finding is explained by cognitive features that characterize individuals with authoritarian traits, as heuristic processing [50] and closeness to experience [52]. Moreover, conservatives show a greater persistence in habitual response patterns, while liberals show greater neurocognitive sensitivity to response conflict and are also more responsive to informational complexity and novelty [54]. In this perspective, it is not surprising that high-RWA participants do not update their behavioral strategy according to external information when they have a positive prior triggered by the high-W expectation (e.g., their investment patterns do not significantly change over time, irrespectively of 50% reciprocation rate), but they are able to update it when they have a negative prior triggered by the low-W expectation. In agreement with our finding, Republicans have been recently found to not adjust their trust behavior to different partisan identities [75].

We believe there are three different (but not mutually exclusive) potential mechanisms explaining our findings: (i) prejudice control/self-regulation, (ii) direct reciprocity (i.e. adjustment to group members’ actual reciprocation behavior) and (iii) indirect reciprocity (i.e. social expectations driven by stereotype content). Firstly, intergroup responses require prejudice control/self-regulation in order to inhibit automatic intergroup biases. Since previous research has shown that low-prejudiced people are more motivated to exert control on their intergroup responses [53] and are also better than conservatives at responding without prejudice [54,55], we expected to find a greater amount of distrustful behavior (i.e., investing more often €1 instead of €5) in high-RWA participants respect to low-RWA ones (but see [97] for results suggesting that conservatives show greater self-control than liberals in cognitive tasks). Since this is not the case, prejudice control alone cannot be responsible for the behavioral patterns we observed in both experiments. Secondly, we could expect that participants adjust to other players’ actual behavior over the trials through reinforcing learning mechanisms [12], in agreement with the direct reciprocity phenomenon [13,14]. However, this explanation does not fully fit with our findings, since it is true only for low-RWA participants interacting with low-W group members (and not high-W ones). Finally, our findings can be interpreted through the social expectation hypothesis, in agreement with the indirect reciprocity phenomenon [13,14]: social groups perceived higher on warmth dimension are going to attract the higher amount of trusting behavior, while the groups perceived lower on warmth dimension are going to be discriminated. Again, this hypothesis does not entirely fit with our findings, since it is true only for high-RWA participants but not for low-RWA ones. We tend to believe that the most reasonable interpretation derives from combining all the hypotheses outlined above. In fact, the willingness to regulate (or not) pre-existing intergroup stereotypes together with social expectations driven by stereotype content and other players’ reciprocation behavior likely interact with one another in determining the observed behavioral pattern. For example, in Experiment 2, low-RWA’s do not discriminate social groups expected to not reciprocate them (low-W) because their egalitarian ideological attitudes help them coping with automatic reliance on group-related stereotypes; moreover they strongly react to defections coming from members of social groups supposed to reciprocate them (high-W). In contrast high-RWA participants’ behavior seems to follow the priors derived from the warmth stereotype when interacting with both low- and high-W social groups. The presence of differential discriminatory tendencies (present for high-W trustees but not for low-W ones) in low-RWA participants could also be due to differences in stereotype content towards the different social groups and/or to the stereotype endorsement degree towards them.

This study has an innovative value because it integrates different research areas ranging from decision-making to social and political psychology. In particular, we suggest that SCM social dimensions seem to be not universally employed in the same way. In fact there are moderating variables (as ideological beliefs) that influence SCM dimensions preferential endorsement (e.g., in the multiple-round TG low-RWA participants distrusted high-W players, while high-RWA participants distrusted low-W ones). Moreover, despite the relationship between warmth and trustworthiness is already well established (see [66,98]) in the context of SCM theory, trust has been previously measured mainly by employing subjective ratings and not ecologically-valid tasks (for exceptions see [29,32,33]).

Importantly, we expand previous knowledge by showing that during social economical interactions (Experiment 2), perceived warmth but not competence, heavily impacts the decision to trust (or not) others. Furthermore, warmth information also guides fast behavioral adjustments during the multiple-round Trust Game. Indeed as the economical interactions proceed, low-authoritarian participants adjust their investment behavior to other groups’ actual reciprocation by investing less in high-W groups’ representatives. Although this result may sound counterintuitive, we believe that differential social expectations about low- and high-W national groups play a key role in explaining this behavioral pattern. In fact, when receiving a neutral feedback during the Trust Game, low-authoritarian participants go on investing on low-W groups (expected to behave unfairly since a lack of warmth equals a lack of reciprocation), but they progressively decide to invest less on high-W groups (supposed to behave fairly since warmth equals to reciprocation). Probably perceived lack of cooperation coming from social groups expected to behave trustworthy is particularly bothering for low-authoritarian players. Interestingly, authoritarian participants kept relying on the reputational priors derived from the warmth stereotype in order to update (or not) their behavioral strategy when facing low- and high-W social groups.

Recently, connectionist models of learning have been employed to study attitude formation and change, with special attention toward intergroup biases. Also in simple neural networks false positive expectancies were corrected by experience, but negative prejudices seem impervious to change [99]. Interestingly, our findings confirm that permeability to external information when learning about others during economical interactions is modulated by the ideological attitude of the participants. In fact, egalitarian participants adapted to others’ behavior depending on their priors but also on others’ actual reciprocation behavior, while authoritarian ones updated their behavioral strategy in the case of negative priors (i.e. low-W groups), but not in the case of positive priors (high-W groups). Interestingly, these behavioral patterns are in agreement with the constructs of *predictive trust* (i.e. a strategic behavior based on the acquired knowledge about national groups) and *altruistic trust* (i.e. a decision based on the believe of the goodwill of that group) [100,101].

We could speculate that in future investigations aimed at measuring how other core ideological attitudes (like social dominance orientation [42] and beliefs in a just world [41] or personality traits related to political ideology (like openness to experience, conscientiousness, neuroticism, intolerance to ambiguity [51,52]) contribute to shape intergroup trust, similar but not identical behavioral patterns could emerge. In particular, we believe that dissimilar findings could be found when investigating SDO. RWA and SDO have been in fact proposed as constructs able to influence prejudice against specific outgroups through different motivational mechanisms [102]: RWA should trigger negative attitudes toward groups perceived as threatening social order (e.g., deviant groups) while SDO should cause negative attitudes toward groups that activate competitiveness over dominance and superiority, for instance socially subordinate groups low in power and status. Furthermore, individuals high in SDO are more likely to obtain leader positions and high-RWA participants tend to act as followers supporting high-SDO leaders. The dyad high SDO leader-high RWA follower makes decisions that are more unethical than those made in role-reversed dyads [103]. On the one hand, these findings suggest that high-SDO participants could show explicit discriminatory tendencies when playing the TG against low-C social groups. On the other hand, the social groups we used in our experiment were probably not able to trigger threat in high-RWA participants that consequently only show explicit discriminatory tendencies towards low-W groups in the multiple-round version of the TG.

A limitation of our study is that we did not explicitly assess participants’ expectations of reciprocity for the different social groups, and thus we can only infer their expectations by analyzing their behavioral patterns. Future research should compare both implicit and explicit participants’ expectations about social partners’ perceived trustworthiness, reciprocity and cooperation.

While we investigated intergroup bias in social interactions by means of the Trust Game, the mechanisms underlying this phenomenon might be involved in other relevant socio-economic decisions, e.g., political decisions about monetary policies, redistribution of funds and payments over sanctions. Future research about social economical interactions in liberal versus conservative participants should take advantage of other paradigms coming from behavioral economics in order to better define the role of the multifaceted psychological features at the base of this ideological divide.

## Supporting information

S1 Text Experiment 1 instructions. (DOCX)

S2 Text Experiment 2 instructions. (DOCX)

S3 Text Warmth and competence ratings (Experiment 2). (DOCX)

S1 Table Descriptive information of warmth and competence ratings relative to European Union group members (N = 50, Experiment 1). Significant t-tests indicate the national groups are rated as ambivalent stereotype groups.
(DOCX)

S1 Fig Mean ratings of warmth and competence for all 44 subjects (Experiment 2). Error bars represent 95% CIs. *** *p* < .001, ** *p* < .01, n.s. *p* > .05.
(TIF)
