# Correction: Right-wing authoritarianism and stereotype-driven expectations interact in shaping intergroup trust in one-shot vs multiple-round social interactions

**DOI:** 10.1371/journal.pone.0191811

**Published:** 2018-01-19

**Authors:** Giorgia Ponsi, Maria Serena Panasiti, Salvatore Maria Aglioti, Marco Tullio Liuzza

The following information is missing from the Funding section: This study was supported by the PRIN grant (Progetti di Ricerca di Rilevante Interesse Nazionale, Edit. 2015, Prot. 20159CZFJK), by the H2020-SESAR- 2015–1 (MOTO: The embodied reMOte TOwer, Project Number 699379), by a research grant to Marco Tullio Liuzza from the Swedish Research Council (2016–02018), and from the Lars Hierta Memorial Foundation (FO20160386).

An affiliation for the fourth author is missing. Marco Tullio Liuzza is also affiliated with the Department of Psychology, Stockholm University, Stockholm, Sweden.
