# Supplementary material for: Right-wing authoritarianism and stereotype-driven expectations interact in shaping intergroup trust in one-shot vs multiple-round social interactions
Source: PLoS One. 2017 Dec 28;12(12):e0190142. doi: 10.1371/journal.pone.0190142 (PMC5746237; doi:10.1371/journal.pone.0190142)
Supplement: S1 Text — (DOCX) [file pone.0190142.s001.docx]

Supporting Information

**Right-wing authoritarianism and stereotype-driven expectations interact in shaping intergroup trust in one-shot vs multiple-round social interactions**

Giorgia Ponsi*, Maria Serena Panasiti*, Salvatore Maria Aglioti and Marco Tullio Liuzza

*****Corresponding Authors

E-mails: [giorgia.ponsi@uniroma1.it](mailto:giorgia.ponsi@uniroma1.it) (GP), [mariaserena.panasiti@uniroma1.it](mailto:marcotullio.liuzza@uniroma1.it) (MSP)

**S1 Text. Experiment 1 Instructions**

Welcome! During this survey you will take part in an economic game with people belonging to other European Countries. In this game, there are two roles: the investor (i.e., the player who chooses how much money to invest) and the trustee (i.e., the player who receives the invested money). During each trial, the investor will be endowed with €1 and he/she may decide whether to invest €0 or €1 in the trustee. Once invested, money will be quadrupled (e.g., an investment of €0 won’t give any financial return, while an investment of €1 will give a financial return of €4). The trustee will have to decide whether to keep for him/herself the entire amount of money or to reciprocate trust by returning half of the sum (e.g., €2 in the case of an original investment of €1). You will play the role of Investor. You have to decide whether to trust or not the other players. The other players have previously made a series of hypothetical choices recorded in our database; in particular, they recorded what they would have done if they had received an investment of €1. In addition to the basic compensation of €1 (for which you will be given a first code), you will receive an additional compensation on the basis of a random extraction of one of the trials of the economic game. If it will be extracted a trial in which you decided to not invest, you will be given a second code in order to gain extra €1, for a total sum of €2 (basic compensation plus second code). If it will be extracted a trial in which you invested on the trustee and the trustee reciprocated you, in addition to the second code, you will be given a third code in order to receive extra €1, for a total sum of €3 (basic compensation plus second code plus third code). In the case of the extraction of a trial in which you invested in the trustee but he/she did not reciprocate your trust, your gain will be of €1 (basic compensation). The extracted trial will determine the final compensation of both you and the partner you were interacting with. Remember to come back to CrowdFlower in order to insert the codes and receive your compensation.
