# Supplementary material for: Right-wing authoritarianism and stereotype-driven expectations interact in shaping intergroup trust in one-shot vs multiple-round social interactions
Source: PLoS One. 2017 Dec 28;12(12):e0190142. doi: 10.1371/journal.pone.0190142 (PMC5746237; doi:10.1371/journal.pone.0190142)
Supplement: S2 Text — (DOCX) [file pone.0190142.s002.docx]

Supporting Information

**Right-wing authoritarianism and stereotype-driven expectations interact in shaping intergroup trust in one-shot vs multiple-round social interactions**

Giorgia Ponsi*, Maria Serena Panasiti*, Salvatore Maria Aglioti and Marco Tullio Liuzza

*****Corresponding Authors

E-mails: [giorgia.ponsi@uniroma1.it](mailto:giorgia.ponsi@uniroma1.it) (GP), [mariaserena.panasiti@uniroma1.it](mailto:marcotullio.liuzza@uniroma1.it) (MSP)

**S2 Text. Experiment 2 Instructions**

Welcome! During this experiment you will take part in an economic game with people coming from Italy, Greece and Germany. In this game, there are two roles: the investor (i.e., the player who chooses how much money to invest) and the trustee (i.e., the player who receives the invested money). During each trial, the investor will be endowed with €5 and he/she may decide whether to invest €1 or €5 in the trustee. Once invested, money will be tripled (e.g., an investment of €1 will give a financial return of €3, while an investment of €5 will give a financial return of €15). The trustee will have to decide whether to keep for him/herself the entire amount of money or to reciprocate trust by returning half of the sum (e.g., €1.5 in the case of an original investment of €1 or €7.5 in the case of an original investment of €15). You will play the role of Investor. You have to decide whether to trust or not the player you are going to face. The other players have previously made a series of hypothetical choices in our database; in particular, they recorded what they would have done if they had received an investment of €1 or €5. In addition to the basic compensation of €5, you will receive an additional compensation on the basis of a random extraction of one of the trials of the economic game. Remember that the extracted trial will determine the final compensation of both you and the partner you were interacting with.
