# Supplementary material for: Right-wing authoritarianism and stereotype-driven expectations interact in shaping intergroup trust in one-shot vs multiple-round social interactions
Source: PLoS One. 2017 Dec 28;12(12):e0190142. doi: 10.1371/journal.pone.0190142 (PMC5746237; doi:10.1371/journal.pone.0190142)
Supplement: S3 Text — (DOCX) [file pone.0190142.s003.docx]

Supporting Information

**Right-wing authoritarianism and stereotype-driven expectations interact in shaping intergroup trust in one-shot vs multiple-round social interactions**

Giorgia Ponsi*, Maria Serena Panasiti*, Salvatore Maria Aglioti and Marco Tullio Liuzza

*****Corresponding Authors

E-mails: [giorgia.ponsi@uniroma1.it](mailto:giorgia.ponsi@uniroma1.it) (GP), [mariaserena.panasiti@uniroma1.it](mailto:marcotullio.liuzza@uniroma1.it) (MSP)

**S3 Text. Warmth and competence ratings (Experiment 2)**

We tested whether the three selected national groups differed in the hypothesized dimensions by entering the ratings into a Repeated Measures 2 (Social Dimensions: Warmth, Competence) x 3 (National Groups: Italians, Greeks, Germans) ANOVA. As predicted, we found a statistically significant Social Dimension x National Group interaction (*F*(2,86) = 70.75, *p* < .001). Regarding the differences between groups, pairwise Bonferroni-corrected comparisons showed that Italians were perceived as warmer (mean = 10.90) than Greeks (mean = 9.72, *t*(43) = -3.62, *p* = .001) and Germans (mean = 7.35, *t*(43) = -6.76, *p* < .001). Also, Greeks were perceived as warmer than Germans (*t*(43) = -4.71, *p* < .001). In terms of competence, Italians (mean = 8.27) did not differ from Greeks (mean = 8.17, *t*(43) = -0.31, *p* > .250) but both groups were rated less competent than Germans (mean = 11.72, *t*s >8.11, *ps* < .001). Concerning the within-group differences, pairwise Bonferroni-corrected comparisons showed that Germans were perceived as more competent than warm (T(43) = -8.60, *p* < .001), Greeks were perceived as more warm than competent (*t*(43) = 4.48, *p* < .001) and Italians were perceived as more warm than competent (*t(*43) = 7.28, *p* < .001) (see S1 Fig).

Such a pattern of results suggest that Germans fall in the Envy (low warmth, high competence) and Greeks in the Pity (high warmth, low competence) quadrants. Interestingly, Italians were not perceived as more competent than Greeks as expected by in-group favoritism phenomenon [1] and ratings towards them fall more into the Pity than into the in-group/Pride (high warmth, high competence) quadrant. This result is consistent with a study on a European sample that clustered Italians along with Greeks, Portuguese, Irish people and Spaniards in the Pity quadrant; Europeans’ ratings tended not to identify high competence-high warmth groups within Europe [2]. Also when European national groups have to rate their in-group they tended to show nothing but a slight bias in warmth ratings [2], which is exactly what we found in Experiment 2.

**References**

1. Brewer MB. The Psychology of Prejudice: Ingroup Love and Outgroup Hate? Journal of Social Issues. 1999;55: 429–444. doi:10.1111/0022-4537.00126

2. Cuddy AJC, Fiske ST, Kwan VSY, Glick P, Demoulin S, Leyens J-P, et al. Stereotype content model across cultures: towards universal similarities and some differences. The British journal of social psychology. 2009;48: 1–33. doi:10.1348/014466608X314935
