# Supplementary material for: Right-wing authoritarianism and stereotype-driven expectations interact in shaping intergroup trust in one-shot vs multiple-round social interactions
Source: PLoS One. 2017 Dec 28;12(12):e0190142. doi: 10.1371/journal.pone.0190142 (PMC5746237; doi:10.1371/journal.pone.0190142)
Supplement: S1 Table — Significant t-tests indicate the national groups are rated as ambivalent stereotype groups. (DOCX) [file pone.0190142.s004.docx]

Supporting Information

**Right-wing authoritarianism and stereotype-driven expectations interact in shaping intergroup trust in one-shot vs multiple-round social interactions**

Giorgia Ponsi*, Maria Serena Panasiti*, Salvatore Maria Aglioti and Marco Tullio Liuzza

*****Corresponding Authors

E-mails: [giorgia.ponsi@uniroma1.it](mailto:giorgia.ponsi@uniroma1.it) (GP), [mariaserena.panasiti@uniroma1.it](mailto:marcotullio.liuzza@uniroma1.it) (MSP)

**S1 Table.** Descriptive information of warmth and competence ratings relative to European Union group members (N = 50, Experiment 1). Significant t-tests indicate the national groups are rated as ambivalent stereotype groups.

|  | **Warmth** | | | **Competence** | | | **Pairwise t-test**  **(two-sided, df = 49)** | | |  |
| --- | --- | --- | --- | --- | --- | --- | --- | --- | --- | --- |
|  | **M** | **(SD)** | **(SE)** | **M** | **(SD)** | **(SE)** | **t** | **p** |  | |
| Austrian | 8.84 | 3.39 | 0.48 | 10.90 | 3.51 | 0.50 | -2.84 | 0.01 |  | |
| Belgian | 9.64 | 3.03 | 0.43 | 9.80 | 3.48 | 0.49 | -0.24 | 0.81 |  | |
| British | 9.34 | 3.84 | 0.54 | 10.42 | 4.11 | 0.58 | -1.44 | 0.16 |  | |
| Bulgarian | 8.32 | 3.11 | 0.44 | 7.96 | 3.56 | 0.50 | 0.58 | 0.56 |  | |
| Croatian | 9.28 | 2.59 | 0.37 | 8.08 | 3.21 | 0.45 | 2.42 | 0.02 |  | |
| Cypriot | 9.70 | 3.22 | 0.45 | 7.62 | 3.06 | 0.43 | 3.57 | 0.00 |  | |
| Czech | 8.86 | 3.05 | 0.43 | 8.70 | 3.25 | 0.46 | 0.25 | 0.80 |  | |
| Danish | 8.86 | 3.25 | 0.46 | 10.54 | 3.59 | 0.51 | -2.34 | 0.02 |  | |
| Dutch | 9.90 | 3.16 | 0.45 | 9.36 | 3.34 | 0.47 | 0.87 | 0.39 |  | |
| Estonian | 8.70 | 3.11 | 0.44 | 9.36 | 3.28 | 0.46 | -1.04 | 0.31 |  | |
| Finnish | 9.02 | 3.11 | 0.44 | 10.32 | 3.55 | 0.50 | -2.03 | 0.05 |  | |
| French | 9.06 | 4.11 | 0.58 | 8.86 | 3.98 | 0.56 | 0.24 | 0.82 |  | |
| German | 7.96 | 3.94 | 0.56 | 11.80 | 4.08 | 0.58 | -5.16 | 0.00 |  | |
| Greek | 11.06 | 3..25 | 0.46 | 6.54 | 3.88 | 0.55 | 6.11 | 0.00 |  | |
| Hungarian | 9.02 | 2.91 | 0.41 | 8.50 | 3.44 | 0.49 | 0.92 | 0.36 |  | |
| Irish | 10.48 | 2.94 | 0.42 | 8.60 | 3.65 | 0.52 | 2.65 | 0.01 |  | |
| Italian | 12.42 | 3.54 | 0.50 | 6.40 | 4.50 | 0.64 | 8.00 | 0.00 |  | |
| Latvian | 8.76 | 3.49 | 0.49 | 9.28 | 3.19 | 0.45 | -0.80 | 0.43 |  | |
| Lithuanian | 8.48 | 3.41 | 0.48 | 8.76 | 3.11 | 0.44 | -0.45 | 0.66 |  | |
| Luxembourgian | 8.76 | 3.35 | 0.47 | 9.62 | 3.76 | 0.53 | -1.09 | 0.28 |  | |
| Maltese | 10.26 | 3.09 | 0.44 | 8.14 | 3.16 | 0.45 | 3.90 | 0.00 |  | |
| Polish | 9.34 | 3.19 | 0.45 | 8.74 | 3.24 | 0.46 | 0.86 | 0.40 |  | |
| Portoguese | 11.10 | 2.87 | 0.41 | 7.76 | 3.84 | 0.54 | 5.40 | 0.00 |  | |
| Romanian | 8.50 | 3.68 | 0.52 | 7.78 | 3.37 | 0.48 | 1.08 | 0.29 |  | |
| Slovenian | 8.98 | 3.01 | 0.43 | 8.38 | 3.00 | 0.42 | 0.96 | 0.34 |  | |
| Spanish | 12.84 | 2.64 | 0.37 | 6.72 | 3.94 | 0.56 | 8.57 | 0.00 |  | |
| Slovakian | 8.68 | 2.90 | 0.41 | 8.98 | 3.11 | 0.44 | -0.48 | 0.63 |  | |
| Swedish | 9.12 | 3.52 | 0.50 | 10.58 | 3.83 | 0.54 | -1.78 | 0.08 |  | |
