# Supplementary figures and images for: Right-wing authoritarianism and stereotype-driven expectations interact in shaping intergroup trust in one-shot vs multiple-round social interactions
Source: PLoS One. 2017 Dec 28;12(12):e0190142. doi: 10.1371/journal.pone.0190142 (PMC5746237; doi:10.1371/journal.pone.0190142)

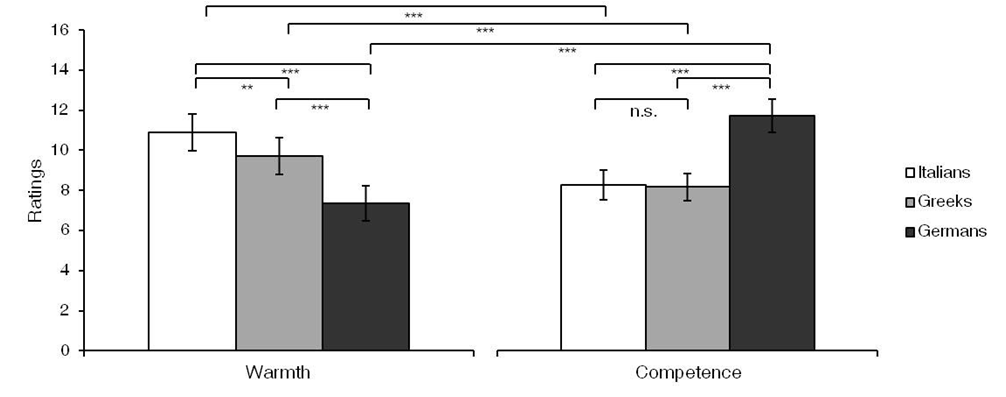

Supplement: S1 Fig — Error bars represent 95% CIs. *** p < .001, ** p < .01, n.s. p > .05. (TIF) [file pone.0190142.s005.tif]
